# Intermittent short ECG recording is more effective than 24-hour Holter ECG in detection of arrhythmias

**DOI:** 10.1186/1471-2261-14-41

**Published:** 2014-04-01

**Authors:** Tijn Hendrikx, Mårten Rosenqvist, Per Wester, Herbert Sandström, Rolf Hörnsten

**Affiliations:** 1 Family Medicine, Department of Public Health and Clinical Medicine, Umeå University, SE-901 87 Umeå, Sweden; 2 Department of Clinical Sciences, Danderyds Sjukhus, Karolinska Institutet, SE-182 88 Stockholm, Sweden; 3 Umeå Stroke Center, Department of Public Health and Clinical Medicine, Umeå University, SE-90187 Umeå, Sweden; 4 Clinical Physiology, Heart Centre and Department of Surgical and Perioperative Science, Umeå University, SE-901 87 Umeå, Sweden

**Keywords:** Arrhythmias, Atrial fibrillation, Electrocardiography, Holter ECG, Intermittent ECG

## Abstract

**Background:**

Many patients report symptoms of palpitations or dizziness/presyncope. These patients are often referred for 24-hour Holter ECG, although the sensitivity for detecting relevant arrhythmias is comparatively low. Intermittent short ECG recording over a longer time period might be a convenient and more sensitive alternative. The objective of this study is to compare the efficacy of 24-hour Holter ECG with intermittent short ECG recording over four weeks to detect relevant arrhythmias in patients with palpitations or dizziness/presyncope.

**Methods:**

*Design*: prospective, observational, cross-sectional study. *Setting*: Clinical Physiology, University Hospital. *Patients*: 108 consecutive patients referred for ambiguous palpitations or dizziness/presyncope. *Interventions*: All individuals underwent a 24-hour Holter ECG and additionally registered 30-second handheld ECG (Zenicor EKG® thumb) recordings at home, twice daily and when having cardiac symptoms, during 28 days. *Main outcome measures*: Significant arrhythmias: atrial fibrillation (AF), paroxysmal supraventricular tachycardia (PSVT), atrioventricular (AV) block II–III, sinus arrest (SA), wide complex tachycardia (WCT).

**Results:**

95 patients, 42 men and 53 women with a mean age of 54.1 years, completed registrations. Analysis of Holter registrations showed atrial fibrillation (AF) in two patients and atrioventricular (AV) block II in one patient (= 3.2% relevant arrhythmias [95% CI 1.1–8.9]). Intermittent handheld ECG detected nine patients with AF, three with paroxysmal supraventricular tachycardia (PSVT) and one with AV-block-II (= 13.7% relevant arrhythmias [95% CI 8.2–22.0]). There was a significant difference between the two methods in favour of intermittent ECG with regard to the ability to detect relevant arrhythmias (P = 0.0094). With Holter ECG, no symptoms were registered during any of the detected arrhythmias. With intermittent ECG, symptoms were registered during half of the arrhythmia episodes.

**Conclusions:**

Intermittent short ECG recording during four weeks is more effective in detecting AF and PSVT in patients with ambiguous symptoms arousing suspicions of arrhythmia than 24-hour Holter ECG.

## Background

Many patients report symptoms of palpitations and dizziness/presyncope. These patients are often referred for 24-hour Holter ECG, although the sensitivity of this investigation for detecting relevant arrhythmias is comparatively low as symptoms in general are transitory, and the patients often are asymptomatic during the investigation [1-4].

Patient-operated intermittent ECG recordings could potentially improve the diagnosis of transitory ECG changes in such patients and may give results comparable to standard external loop event recorders [5,6]. The advantage of such devices compared to standard external loop event recorders is that they are reasonably priced and easy to use, especially as no external electrodes are necessary.

Even when using (handheld or standard external loop) event recorders, episodes of an arrhythmia may be missed as the correlation between symptoms and relevant arrhythmias is often not very strong. In atrial fibrillation (AF), for example, it is known that only one in 10 paroxysms is symptomatic [7]. The European Heart Rhythm Association stated in a 2011 position paper on palpitations that it is especially important to exclude AF as the underlying cause of symptoms in patients with palpitations of unknown origin, as AF is associated with an increased risk of thrombo-embolism [8-10]. Recent studies show that intermittent ECG recording with both regular and symptomatic registrations detects more episodes of silent AF in patients with known paroxysmal atrial fibrillation compared with 24-hour Holter ECG [11] and improves the detection of previously unknown asymptomatic paroxysmal atrial fibrillation (AF) in post-stroke patients [12].

The objective of this study is to compare the efficacy of short intermittent ECG registrations with 24-hour Holter ECG, in detecting relevant arrhythmias in patients reporting symptoms of palpitations and dizziness/presyncope.

## Methods

### Design, study population and setting

**Figure 1:**
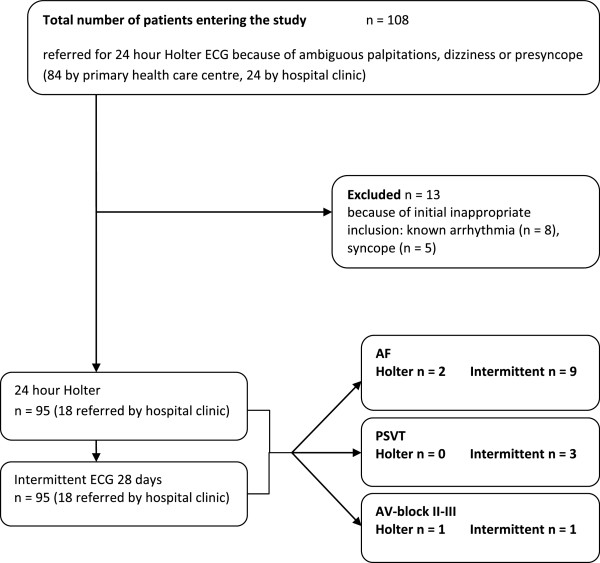
Study flowchart.

In this prospective, observational, cross-sectional study 108 consecutive patients with symptoms of ambiguous palpitations or dizziness/presyncope, referred to the Department of Clinical Physiology, Norrland University Hospital, Umeå, for 24-hour Holter ECG, were included. (Flowchart, Figure 1). The catchment area in the present study was the county of Västerbotten (population 212 660). The Department of Clinical Physiology is the only centre in Västerbotten County for this type of service. Palpitations were defined as a sensation in which a person is aware of an irregular, hard or rapid heartbeat. Dizziness/presyncope was defined as a sensation in which a person experiences light-headedness, unsteadiness or near-fainting. Exclusion criteria were: known arrhythmia, based on previous history or 12-lead ECG performed at the time of referral; referral for syncope, defined as temporary loss of consciousness; or comorbidity with cognitive or other functional impairments impeding the use of the handheld device. Of 108 patients 84 were referred by a primary health care centre and 24 by a hospital clinic. The study complies with the Declaration of Helsinki and was approved by the Regional Ethical Review Board, Umeå (Dnr 07-051M). All participating patients gave written informed consent.

### Intervention

Holter recordings were performed using a standard recording unit (Breamer DL700, Breamer Inc. Burnsville, MN, USA). Holter recordings were automatically analysed by a PC-based Holter system (Aspect Holter System, GE Healthcare, Stockholm, Sweden).

Patients were asked to additionally perform 30-second intermittent handheld ECG (Zenicor EKG® thumb, Stockholm, Sweden) registrations at home for 28 days, twice daily, morning and evening, and when having cardiac symptoms, resulting in a total registration time of approximately half an hour. Intermittent recordings started on the same day as the 24-hour Holter ECG recording.

Handheld ECG registrations and Holter registrations were evaluated separately by two investigators with expertise in arrhythmology who were blinded to the result of the other method. In case of diagnostic uncertainty, a consensus was reached by the two investigators together with a cardiologist.

### The handheld device

Zenicor EKG® thumb is a handheld device which via both thumbs registers a bipolar extremity lead I ECG for 30 seconds. After each registration the recording is transmitted by the patient via the built-in mobile device (SIM card) to a web-based central database. Symptomatic episodes can be marked with a button. In the database these symptomatic registrations are highlighted. The ability to give the correct diagnosis of AF compared to 12-lead ECG has shown a sensitivity of 96% and a specificity of 92% [13]. A detailed technical description of the device and its performance is published elsewhere [13].

Information on age; sex; smoking; earlier cardiac ultrasound, carotid doppler and ECG; CHA_2_DS_2_-VASc risk factors; amount and duration of earlier episodes of palpitations and the presence of dizziness was collected in a Case Report Form (CRF) in the web-based central database.

### Outcome measures

Significant arrhythmias were defined as: atrial fibrillation (AF) ≥30 seconds and AF defined as irregular heart rhythm without distinct sinus p-waves; paroxysmal supraventricular tachycardia (PSVT) ≥30 seconds, defined as regular rhythm, with supraventricular extrasystoles (SVES) in series, >120 beats/minute; atrioventricular (AV) block II–III; sinus arrest (SA) >2.5 seconds; wide complex tachycardia (WCT) with a QRS width of >120 ms and with a heart rate >100 beats/minute and at least 3 wide QRS complexes after each other. Patients with detected arrhythmias were referred for treatment in accordance with national guidelines.

### Statistics

Continuous variables were presented with median and interquartile range (IQR), except for age and heart rate which were presented with mean, standard deviation (SD) and range. Categorical variables were presented with count and percentage and, where appropriate, a 95% confidence interval. To test the hypothesis that there is a statistical difference in the efficacy of intermittent ECG recordings compared to 24-hour continuous Holter monitoring in detecting relevant arrhythmias, we used McNemar’s test for paired proportions. To evaluate possible differences in age, the number of registrations and the amount and duration of previous episodes with palpitations between individuals with and without detected AF, the Mann-Whitney U test was used. To evaluate the relation between history of palpitations, sex and CHA_2_DS_2_-VASc categories and the detection of AF, the Pearson Chi-square test was used. SPSS Statistics 19.0 (IBM Corporation, Route 100 Somer, NY 10589) was used for all calculations. The level of significance was set at 0.05, two-sided. The study was dimensioned to detect clinically relevant discrepancies between the two methods based on the following population effect size: in 80% of the patients, both methods will classify a patient as negative for a relevant arrhythmia and in another 3% both methods will classify a patient as positive. A discrepancy between the methods was assumed in the population as follows: in 14% of all patients only one test will show an outcome of positive relevant arrhythmia, while 3% of all patients will show an outcome of positive relevant arrhythmia for the other method. We needed 90 patients to yield a power of 80% with a statistically significant result with alpha of 0.05 (two-tailed). Calculating with a drop-out rate of 15% 106 patients needed to be included; the power calculation was performed in SamplePower 2.0.

## Results

### Demographics

Ninety-five patients, 42 men and 53 women with a mean age of 54.1 years, completed registrations. Thirteen of the originally included 108 patients were excluded after initial inappropriate inclusion; they either had a known tachyarrhythmia (n = 8) or suffered from syncope (n = 5). (Flowchart, Figure 1. Demographic data, Table 1). Of these 95 patients, 80 were referred for palpitations and 15 for dizziness/presyncope. 77 patients were referred by a primary health care centre and 18 by a hospital clinic.

**Table 1:** Demographic characteristics of the study population

**Demographic characteristics**			
Total population [n]	95		
Men [n, (%)]	42	(44.2)	
Age [years, mean, SD, (range)]	54.1	±16.4	(21–79)
Primary care referral [n, (%)]	77	(81.1)	
Smoking [n, (%)]	7	(7.4)	
Earlier palpitations, [n, (%)]	80	(84.2)	
Dizziness/presyncope [n, (%)]	15	(15.8)	
Congestive Heart Failure [n, (%)]	0		
Hypertension [n, (%)]	27	(28.4)	
Age ≥65 < 75 [n, (%)]	30	(31.6)	
Age ≥75 [n, (%)]	9	(9.5)	
Diabetes [n, (%)]	1	(1.1)	
Earlier stroke/TIA/TE [n, (%)]	6	(6.3)	
Cardiovascular disease [n, (%)]	8	(8.4)	
CHA_2_DS_2_-VASc = 0 [n, (%)]	19	(20)	
CHA_2_DS_2_-VASc = 1 [n, (%)]	45	(47.4)	
CHA_2_DS_2_-VASc ≥ 2 [n, (%)]	31	(32.6)	
Palpitation episodes last year [n, median, (IQR)]	110	(27.3–176.3)	
Duration palpitation episodes last year [min, median, (IQR)]	10	(3.5–30)	
Patients with symptoms during intermittent ECG [n, (%)]	31	(32.6)	
Patients with symptoms during 24-hour Holter [n, (%)]	40	(42.1)	
Intermittent registrations, total [n, median, (IQR)]	59	(48.5–65)	
Intermittent registrations with symptoms [n, median, (IQR)]	3	(0–9)	
Intermittent registrations with analysable tracing quality [n, median, (IQR)]	57	(45.5–65)	
24-hour Holter registration time with analysable quality of tracing [h.min, median, (IQR)]	24.00	(23.55–24.00)	

IQR = interquartile range; SD = standard deviation; TE = thrombo-embolism; TIA = transient ischemic attack.

All patients were able to perform intermittent registrations. The median number of intermittent ECG registrations was 59 with and interquartile range (IQR) of 48.5– 65. Seventy-one patients (74.7%) completed at least 50 registrations and only eight patients (8.4%) made less than 28 registrations, i.e., half of the stipulated 56 registrations. Holter patients completed their 24-hour recording with the exception of one patient who recorded only 11 hours and eight minutes.

Both handheld and Holter registrations were of good quality. Only 1.6% of handheld registrations (84 of 5229 registrations) and 1.3% of Holter registrations (30 of 2280 hours) were of non-analysable quality.

### Previously experienced palpitations

The characteristics of palpitations experienced within the last 12 months before performing the ECG investigations are presented in Table 1 and 2. No data on the number of episodes of dizziness/presyncope and duration of these episodes were included.

**Table 2:** Demographic and intermittent ECG characteristics of patients with and without AF

**Demographic characteristics**		**AF**			**Non AF**			**P-Value**
Patients [n, (%)]		9	(9.5)		86	(90.5)		
Men [n, (%)]		3	(33.3)		39	(45.3)		0.490
Age [years, mean, SD, (range)]		58.3	±11.5	(28–67)	53.8	±16.8	(21–79)	0.476
Referred for palpitations [n, (%)]		9	(100)		71	(82.6)		0.172
CHA_2_DS_2_-VASc = 0 [n, (%)]		0			19	(22.1)		0.115
CHA_2_DS_2_-VASc = 1 [n, (%)]		5	(55.6)		40	(46.5)		0.605
CHA_2_DS_2_-VASc ≥ 2 [n, (%)]		4	(44.4)		27	(31.4)		0.427
Palpitation episodes last year [n, median (IQR)]		110	(105–115)		110	(25.8–178.8)		0.969
Duration of palpitation episodes [min, median (IQR)]		10	4.3–26.3		10	3.5–30		0.979
**Intermittent ECG characteristics**		**AF**			**Non AF**			**P-Value**
Time to detection [n, (%)]	Day 1	1	(11.1)					
	Day 2–14	5	(55.6)					
	Day 15–28	3	(33.3)					
Heart rate AF [bpm, mean, SD, (range)]		145	±19.0	(120–184)				
Registrations, total [n, median (IQR)]		61	(58–70)		57.5	(46.8–64.8)		0.234
Registrations with symptoms [n, median (IQR)]		9	(2–9)		2.5	(0–8.8)		0.216
Registrations with analysable tracing quality [n, median (IQR)]		61	(54–70)		57	(45–64.8)		0.247
Intermittent registrations with AF [n, median (IQR)]		4	(1–5)					
Symptomatic AF registrations [n, median (IQR)]		2	(0–3)					

AF = atrial fibrillation; IQR = interquartile range; SD = standard deviation.

### Detection of relevant arrhythmias

Analysis of the 24-hour Holter recordings showed AF in two patients and AV-block II in one patient. This resulted in a total of 3.2% relevant arrhythmias [95% CI 1.1–8.9] detected. Nine patients with AF were detected with intermittent ECG. Two of these were the same as those discovered with Holter ECG; one of them had persistent AF. Two AF patients also had episodes with a fast regular rhythm, either atrial flutter or PSVT. Three patients were diagnosed with PSVT and one patient with AV-block II. One additional patient who continued with registrations on his own initiative after day 28 had an AF episode at day 50. As this was not detected within 28 days he was not counted as newly diagnosed AF within the study framework. In total 13.7% relevant arrhythmias [95% CI 8.2–22.0] were detected with intermittent handheld ECG. (Detected arrhythmias, Table 3). The statistical analysis showed a significant difference between the two methods in favour of intermittent handheld ECG recordings with regard to the ability to detect relevant arrhythmias (P = 0.0094).

**Table 3:** Detection of relevant arrhythmias – 24-hour Holter ECG versus intermittent ECG

	**Intermittent ECG positive**	**Intermittent ECG negative**	**Added results**
**24-hour Holter ECG positive**	2	1	3^*^
**24-hour Holter ECG negative**	11	81	92
**Added results**	**13**^†^	82	Total: 95

*24-hour Holter ECG added results. n = 3, 3.2%, [95% CI 1.1–8.9].

^†^Intermittent ECG added results. n = 13, 13.7%, [95% CI 8.2–22.0].

All arrhythmia episodes during Holter were asymptomatic. Forty-four percent of all arrhythmia episodes with intermittent recording were asymptomatic.

### Atrial fibrillation

**Figure 2:**
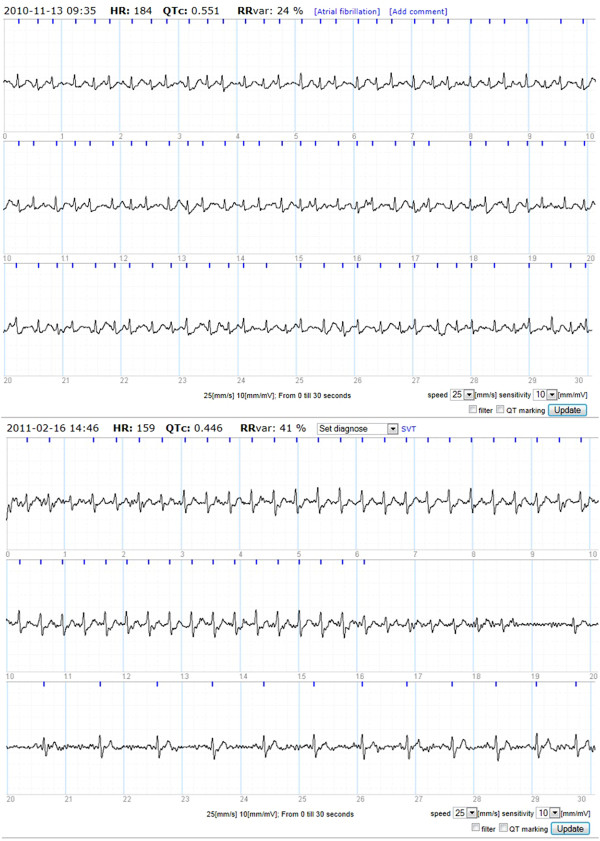
Example of handheld ECG registration of AF and PSVT.

With nine AF patients detected with intermittent handheld ECG AF was the main arrhythmia recorded. (An example of a handheld ECG registration of AF is provided in Figure 2). One AF patient was found on day one and the last AF patient was found day 26. Two AF patients, one of them with persistent AF, were the same as those discovered with Holter ECG. Patients with AF were slightly older and had slightly higher CHA_2_DS_2_-VASc scores compared to those without AF, but these differences were not statistically significant. No statistically significant differences for previous palpitations were observed between the AF and non-AF group. (Characteristics of patients with AF, Table 2). Out of a total of 61 intermittent registrations (median) for AF patients (Mean 62; SD ±17.80; Range 48-89) nine (median) were symptomatic (9/61 = 14.8%). (Table 2). (Mean 7.33; SD ±5.23; Range 0-16). These patients had four registrations (median) that showed AF, (4/61 = 6.6%). (Table 2). (Mean 9.33; SD ±18.34; Range 1-61) All AF patients had a CHA_2_DS_2_-VASc of one or more and were therefore potential candidates for oral anticoagulation treatment.

### Paroxysmal supraventricular tachycardia

PSVT was detected in three patients with handheld ECG with a mean heart rate of 177 bpm (SD ± 18.8, range 154–200). In two patients at least one episode was symptomatic. (An example of a handheld ECG registration of PSVT is provided in Figure 2).

### Atrioventricular-block II

One patient with AV-block II, discovered with Holter, was referred for Holter because of palpitations, the other, discovered with intermittent ECG, because of dizziness/presyncope. None of these patients had symptoms related to the recorded arrhythmia.

## Discussion

### Results

The main finding of this study is that intermittent ECG recording is superior to routine 24-hour Holter ECG in detecting relevant paroxysmal arrhythmias in a patient population reporting symptoms of palpitations, dizziness/presyncope. The novelty in this study compared to other studies searching for paroxysmal arrhythmias is the use of prolonged intermittent recording for four weeks, both regularly twice daily and when having symptoms. To our knowledge, there are no earlier reports comparing brief intermittent long-term ECG with 24-hour Holter ECG in detecting paroxysmal arrhythmias in patients referred for ambiguous cardiac symptoms.

It was already known that the sensitivity of 24-hour Holter recordings for detecting relevant arrhythmias is low but it is still widely used as a routine in primary care and hospital settings. The fact that almost half of the intermittent recordings showing significant arrhythmias were recorded without associated symptoms emphasizes that this method has advantages compared to standard Event recording.

At the same time it should be mentioned that it has been previously estimated that only one in ten paroxysms of AF is symptomatic [7,14]. The much higher rate, about fifty percent, of symptomatic episodes in this study may be explained by the fact that included patients are selected for having ambiguous cardiac symptoms.

### Relevance

The two main arrhythmias detected in this study were AF and PSVT. Occurrence of AF constitutes in itself an independent risk factor for stroke, and with concurrent other risk factors (congestive heart disease, hypertension, age ≥65 years, age ≥75 years, diabetes, earlier stroke/transient ischemic attack (TIA)/thrombo-embolism (TE), vascular disease and female sex (CHA_2_DS_2_-VASc)), this risk is additionally increased [8,15]. It is extremely important to detect and treat AF patients before they suffer a stroke. As stroke risk is the same for symptomatic and non-symptomatic AF patients, [16] this method could also be warranted for screening of asymptomatic patients with higher CHA_2_DS_2_-VASc scores [17-19]. Detection of PSVT is relevant as it can be caused by re-entry tachycardias, potentially curable with ablation. Previous studies have also shown that PSVT patients have a higher risk of developing atrial fibrillation [20] and flutter [21].

The study population consisted of relatively young and healthy patients of whom the majority were referred from Primary Care. However, the fact that all AF patients had a CHA_2_DS_2_-VASc score ≥ 1 indicates that most of these patients could be candidates for oral anticoagulation (OAC) treatment. (Table 2).

### Device and recording protocol

**Figure 3:**
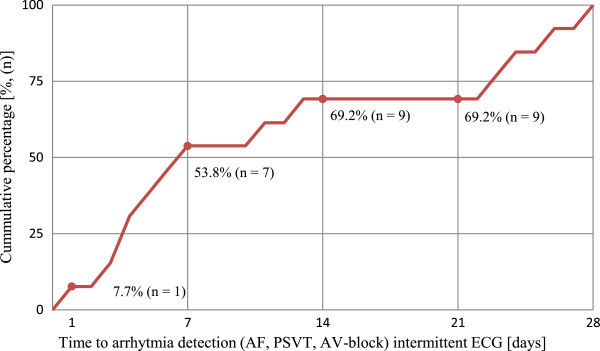
Time to detection (days) of relevant arrhythmias (AF, PSVT and AV-block II) with intermittent handheld ECG recording.

The device is small and seldom limits the mobility of the patients. Registrations are easy to perform. The chosen method gives the possibility for both asymptomatic and symptomatic registrations and allows registration over a long time period, which seems to be important as only two thirds of the recorded arrhythmias occurred within 14 days. (Figure 3, Table 2). An advantage compared to devices used in other studies [5,6] is that the recording is transmitted by the patient via the built-in mobile device (SIM card) directly to a web-based central database and can be analysed online.

### Compliance

Compliance with the intermittent handheld ECG method was high. The 95 included patients had a median of 59 registrations (IQR 48.5–65), which is more than two registrations a day for 28 days. The high compliance rate using this method indicates that it is a feasible method for screening in larger patient populations.

### Limitations

With the handheld device no registrations are made during physical exertion, sleep or syncope. Other devices, e.g. patch-based appliances with the possibility of long-term continuous recording, are most likely an even better alternative than devices for short intermittent recording, as these would not miss episodes during physical exertion, sleep, or syncope [22]. Smart phone based applications can, in the light of their high accessibility, also be expected to become useful tools for screening and monitoring of AF and other paroxysmal arrhythmias [23-25].

The handheld device only records lead I, which sometimes makes it difficult to distinguish atrial flutter from sinus rhythm or a regular supraventricular tachycardia. This might have resulted in underdetection of atrial flutter [13].

Registrations are only 30 seconds. If there is an arrhythmia like AF only at the beginning or at the end of the registration we cannot be certain of how long this episode actually lasted. The AF definition used by the European Society of Cardiology in its Guidelines from 2010: ‘Any arrhythmia that has the ECG characteristics of AF and lasts sufficiently long for a 12-lead ECG to be recorded, or at least 30 s on a rhythm strip, should be considered as AF’, is based on consensus and the clinical implication for risk of stroke is as yet not fully understood [26].

### Generalizability

All patients referred to the Department of Clinical Physiology for ambiguous palpitations or dizziness/presyncope were included consecutively. Except for known arrhythmia, referral for syncope, or comorbidity with cognitive or other functional impairments impeding the use of the handheld device, no other exclusions were made. The Department of Clinical Physiology is the only clinic that performs Holter ECG in the province of Västerbotten, Northern Sweden therefore selection bias is limited. Other studies investigating palpitations looking at a general population show quite similar characteristics for age, sex and comorbidity [1,10,27,28].

## Conclusions

Short intermittent ECG recording, at regular time intervals and when having symptoms, during a four-week period, is more effective than routine 24-hour Holter ECG in detecting AF and PSVT in patients with palpitations. The fact that half of the AF episodes were asymptomatic implies that even Event recording has its limitations in this patient category.

## Abbreviations

AF: Atrial fibrillation; AV: Atrioventricular; CI: Confidence interval; ECG: Electrocardiogram; PSVT: Paroxysmal supraventricular tachycardia; SA: Sinusarrest; WCT: Wide complex tachycardia; WPW: Wolf-Parkinson-White.

## Competing interests

This study was supported by grants from Umeå University Hospital and Vinnova.

Zenicor Medical Systems AB, Stockholm, has provided handheld ECG devices (Zenicor EKG® thumb) at a reduced price.

## Authors’ contributions

TH took part in conceiving and designing the research, acquired data, analysed and interpreted data, performed statistical analysis and drafted and revised the paper. He is guarantor. MR conceived and designed the research, handled funding and supervision and made critical revisions of the manuscript. PW made critical revisions of the manuscript. HS took part in conceiving and designing the research, handled funding and supervision and made critical revisions of the manuscript. RH took part in conceiving and designing the research, acquiring data, analysing and interpreting data and revising the manuscript. All authors read and approved the final manuscript.

## Pre-publication history

The pre-publication history for this paper can be accessed here:

http://www.biomedcentral.com/1471-2261/14/41/prepub
